# Surveillance Analysis and Sample Size Explorer (SASSE): Learning How to Plan Disease Surveillance in Wildlife

**DOI:** 10.1002/ece3.71991

**Published:** 2025-08-15

**Authors:** Lauren Smith, Joshua Hewitt, Aaron Westmoreland, Grete Wilson‐Henjum, Kezia Manlove, Kim M. Pepin

**Affiliations:** ^1^ National Wildlife Research Center Wildlife Services, Animal and Plant Health Inspection Service, United States Department of Agriculture Fort Collins Colorado USA; ^2^ Department of Wildland Resources and Ecology Center Utah State University Logan Utah USA

**Keywords:** detection, prevalence, sample design, surveillance objectives, surveillance planning, wildlife disease

## Abstract

Wildlife disease surveillance helps in protecting public health, agriculture, and biodiversity. Planning effective surveillance involves strategic methods for identifying an effective sampling design for a program's objectives. Gaps in existing standards and complexity for wildlife surveillance justify a need for tools that can build statistically based intuition in wildlife professionals when designing surveillance systems. To address this need, we present the use of plug‐and‐play tools, specifically our surveillance analysis and sample size explorer (SASSE), to allow wildlife professionals to build intuition about the role of sample size vis‐à‐vis sampling design and diagnostic test performance in wildlife systems. SASSE uses open‐source software (R, R Shiny) to design an interactive, module‐based teaching tool to cover key surveillance objectives, including detection, prevalence, and epidemiological dynamics. Our tool fills the following gaps: (1) allows a broad audience to apply statistical sample size theory for designing disease surveillance, and (2) provides a simple statistical tool for addressing challenges with disease surveillance design in wildlife populations. Thus, the tool we present here can be used readily to identify efficient sampling designs for a surveillance objective across a wide variety of settings.

## Graphical abstract

Surveillance of wildlife disease is important and often involves strategic methods to identify an effective design for a program's objectives that accounts for ecological and epidemiological complexities in the system. We present an interactive teaching tool, Surveillance Analysis and Sample Size Explorer (SASSE), to allow wildlife professionals to build statistically based intuition on how to design surveillance for a given objective in complex wildlife systems.

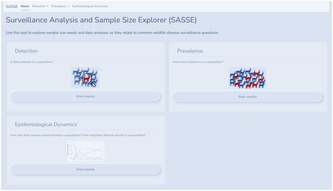

## 1 Introduction

Disease surveillance of wildlife populations is important for controlling and preventing the risk of zoonotic spillover to humans (Daszak et al. 2000; Lloyd‐Smith et al. 2009; Miller et al. 2017; Plowright et al. 2017; Cunningham et al. 2017), the risk of spillover to livestock (Artois et al. 2001; Miller et al. 2017), and the resulting agricultural economic impact (Shwiff et al. 2016), as well as the risk to biodiversity and conservation of wildlife species themselves (Daszak et al. 2000; Artois et al. 2009; Cardoso et al. 2022). Efficient surveillance requires sample design methods that fit the surveillance objectives and ecological processes at hand (Nusser et al. 2008; FAO 2014). Developing effective designs requires practitioners to integrate information about which and how many individuals to sample, how well the diagnostic tests perform, and the timing of sampling relative to the epidemiological characteristics of the host–pathogen system (Nusser et al. 2008; Cameron et al. 2014). Implementing the design can be challenging and usually requires on‐the‐ground decision‐making in real‐time to choose between sites or sample sizes that will best approximate the intended design. These decisions can be guided by statistical sampling theory, but that may be infeasible to implement while conducting surveillance. One solution is to develop training materials that teach the user to develop intuition for decision‐making in the field that can deliver the best surveillance data for real‐world challenges that arise.

Surveillance and monitoring for disease in wildlife may involve opportunistic surveillance arising from dead, sick, or injured animals, or animals captured for other purposes; or targeting specific locations, individuals, or populations (Artois et al. 2009; Clow et al. 2019). Hunter harvest or wildlife removal sampling is a common form of opportunistic disease surveillance; however, detection biases can occur if harvest is disproportionately concentrated in specific sexes, ages, locations, seasons, or health groups (Nusser et al. 2008; Miller et al. 2013; Belsare et al. 2020; Wilber et al. 2020). These detection biases can be alleviated through careful consideration of sample designs for a given situation.

There is a robust methodology developed for designing surveillance in livestock populations (e.g., Martin et al. 2007). This methodology can be expanded to develop simple tools for guiding surveillance design decisions in wildlife. A key assumption in sample size theory applied to livestock is that host abundance is constant and known, which is usually not the case for wildlife (Artois et al. 2001; Walton et al. 2016). Because of the free‐roaming nature and complex spatial and social ecology of many wildlife host species, methods for adapting livestock approaches to wildlife surveillance design are needed. Another difference is that the sensitivity of diagnostic assays can be lower and more variable in wildlife relative to livestock, especially for serological assays, and is often optimized in livestock species but unknown in wildlife species (Gilbert et al. 2013). Thus, adapting livestock sampling design theory for wildlife applications needs to account for differences related to sampling biases, diagnostic uncertainty, and uncertainty in abundance, which together influence prevalence estimates.

In this paper, we introduce Surveillance Analysis and Sample Size Explorer (SASSE), an interactive tool for wildlife professionals to bridge the gap in applying standard sampling design statistics to wildlife‐specific contexts. Here, we present a web‐based plug‐and‐play tool with related teaching materials for addressing broad surveillance design questions of “when,” “where,” and “how many” for the surveillance objective. Each module of SASSE has two separate applications aimed to: (1) Guide sampling design (power analysis): help the user develop intuition for choosing an appropriate sample size in the field, and (2) Evaluate surveillance data (data inference): provide a rapid evaluation tool for interpreting uncertainty in epidemiological inferences from surveillance data. The tool accounts for the context of multiple host–pathogen systems and sampling constraints. We package the statistical theory and inference into an interactive application that does not require a technical background for use and corresponding lecture notes to make our tool accessible to all users, regardless of statistical background and programming skills. In this format, our tool can also be used for education for wildlife students or professionals to teach rigorous methodology for designing wildlife populations or disease research, and operational activities.

## 2 Application Overview

We developed an R Shiny (Chang et al. 2024; R Core Team 2024) Application and corresponding set of statistical models to guide surveillance design. Accompanying lecture notes and exercises provide documentation and tutorial materials for the application, building epidemiological intuition for students, researchers, and wildlife professionals. Our tool is founded in the idea of connecting surveillance objectives to appropriate surveillance designs. The tool focuses on three surveillance objectives: detection (Understanding Whether a Pathogen Is Present at a Given Site Sampled), prevalence (Understanding What Proportion of Individuals Are Infected or Have Been Exposed to a Pathogen at a Given Site Sampled), and epidemiological dynamics (Understanding Transmission Dynamics of the Pathogen Through Time at a Given Site Sampled) (Table 1). Some interpretation for evaluating seasonal changes in detection and prevalence is also included. The following sections describe the tool's support for each objective.

**TABLE 1:** Surveillance objectives overview.

Metric	Scale	Example surveillance questions
Detection	Individual^a^ Population	Which individuals are most at risk?^a^ Is there disease in a population?
Prevalence	Population	What proportion of the population is affected?
Epidemiological dynamics	Population	What are the rates of infection and how do they change over time? Is the system predictable? What are the ecological drivers?
Seasonality^b^	Population	How frequent are outbreaks?

*Note:* Topic overview table used for introducing SASSE learning modules. This includes a broad description of the surveillance objectives and example surveillance questions that could be answered with each module.

^a^ The detection module is unique in its ability to answer questions at the individual scale. The sampling stratum component (described in Section 3: Module Workflow) allows the user to subset samples into categories that are relevant to their system, which may include individual‐level characteristics such as age or sex.

^b^ Seasonality module is not yet included in the application interface but is mentioned in lecture material.

### 2.1 Shiny App

Our application, the surveillance analysis and sample size explorer (SASSE), was developed using R Shiny, a common platform used for interactive dashboards, web applications, and analysis tools. R Shiny was used because (1) our statistical models were written in the R programming language; (2) R is open source and commonly used in the wildlife field, which opens the door for other wildlife professionals to collaborate in future versions; and (3) R Shiny provides pre‐existing infrastructure for connecting R inputs and outputs with aesthetic and user‐friendly web applications. SASSE aims to translate our mathematical models that answer surveillance questions into a visually appealing, user‐friendly format so that our broader wildlife audience can use and interpret the material without a statistical or programming background. This is achieved by providing interactive inputs and dynamic graphical outputs in each module; the user can change inputs and observe how changes are reflected in the outputs to deduce various relationships and interpretations of surveillance planning metrics.

**FIGURE 1:**
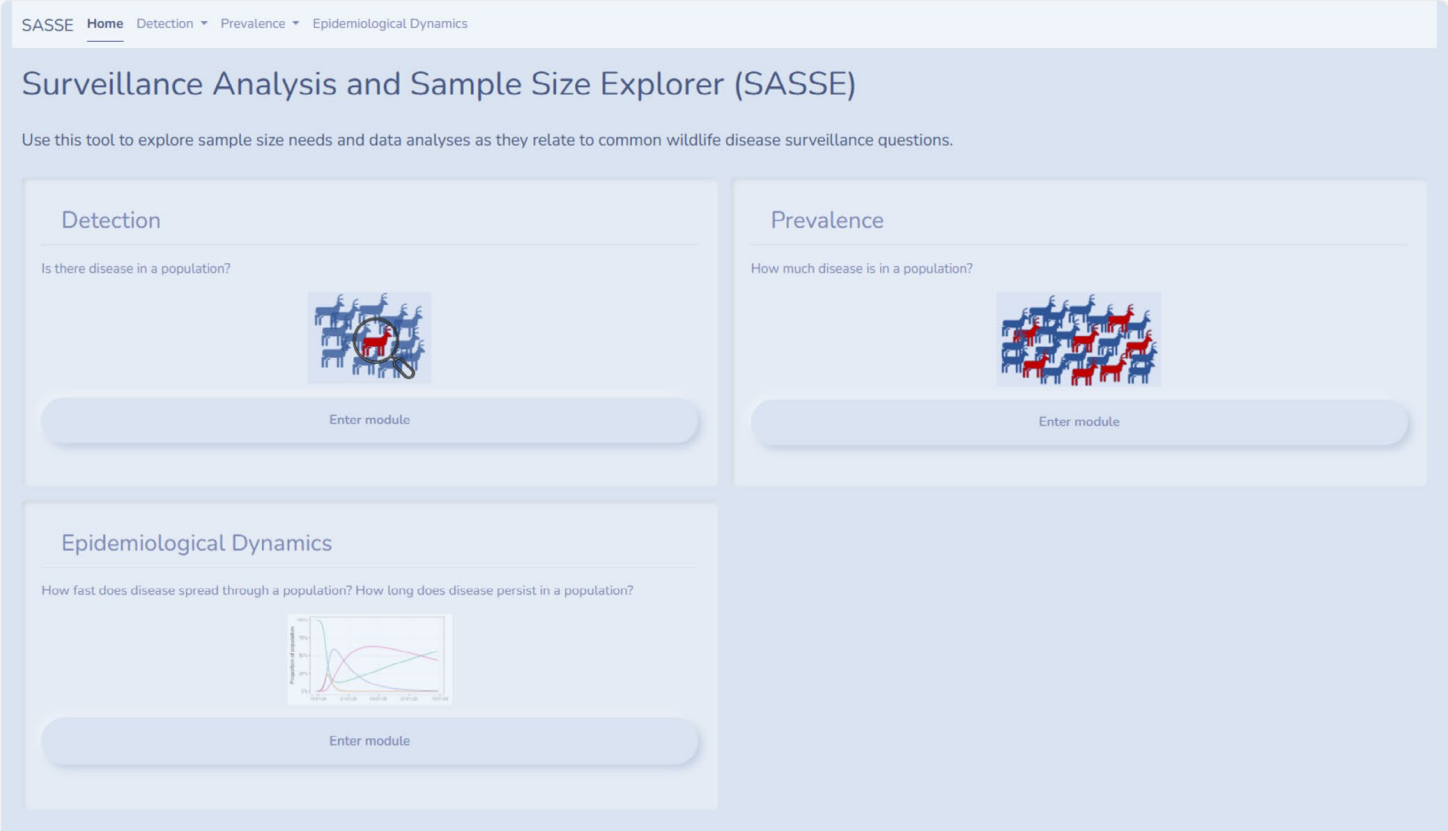
SASSE modular interface. Landing page view of the SASSE web app, showing the three modules: detection, prevalence, and epidemiological dynamics.

SASSE is built with a modular interface, with the landing page displaying one module for each surveillance metric (Figure 1). Each module has a graphic and a surveillance objective question to provide context as to what it covers. Users can enter each module by selecting the “Enter module” button and then navigate through the modules using the buttons at the bottom of each page. Section 3, the Module Workflow, walks through the content of each module in detail. All modules are built around statistical models; the interface itself only shows inputs and outputs, but the details of each model are provided here and on GitHub (archived: https://doi.org/10.5281/zenodo.16647143 public link: https://github.com/deerdisease/sasse SASSE link: https://deerdisease.shinyapps.io/Wildlife‐surveillance‐design‐tools/).

### 2.2 Statistical Model

Under SASSE's hood are statistical models that address each surveillance objective. The detection and prevalence modules each contain an analysis model for interpreting the uncertainty in the epidemiological inference and a power study model for guiding surveillance planning decisions and developing intuition for spontaneous decision‐making while implementing surveillance designs. The epidemiological dynamics module has a separate stand‐alone model. Definitions related to the three models can be found in the glossary (Table 2).

**TABLE 2:** Glossary.

Module(s)	Model(s)	Input/Output	Metric	Definition
Detection & prevalence	Data analysis	Input	*N* samples	Number of surveillance samples at a site
			*N* positives	Number of positive samples at a site
	Data analysis & power study	Input	Sensitivity	True positive detection rate
			Specificity	True negative detection rate
			Prior presence	Expected chance of disease presence
			Prior prevalence range	Potential range of prevalence if pathogen is present
	Power study	Input	True prevalence	Percent of individuals in the population that are infected with a pathogen at the time of sampling
Detection	Data analysis	Output	Disease freedom probability	Chance the population is free from disease
			Disease presence probability	Chance disease exists in the population
			Prevalence upper bound	Upper boundary for what prevalence will not exceed if there is not 100% disease freedom in a population
	Power study	Output	Disease freedom accuracy	Percent of time studies with stated assumptions declare the population is free from disease when there actually is disease
			Disease presence accuracy	Percent of time studies with stated assumptions declare the population has disease when there actually is disease
Prevalence	Data analysis	Output	Apparent prevalence	Percent of individuals in the sample infected with the pathogen
			Model estimate of true prevalence	Model estimate of the percent of individuals in the population that are infected with a pathogen at the time of sampling
			Credible interval	Bayesian confidence interval
	Power study	Output	Credible interval coverage probability	Chance that a study with the stated assumptions will have a credible interval that covers the true prevalence
			Average credible interval width	Average width of the credible interval
Epidemiological dynamics	SIBR	Input	Avg infectious days	Average number of days individuals are infectious with detectable pathogen
			Avg recovery days	Average number of days it takes to clear all pathogen once antibodies are detectable
			Avg waning days	Average number of days infection takes for antibodies to be cleared and the host to be susceptible to infection again
			R0	Basic reproduction number of the pathogen (average number of transmissions for an infectious individual in a completely susceptible population)
			Susceptible	Number of individuals susceptible to infection
			Infectious	Number of individuals that shed pathogen and can transmit to others
			Broadly recovered	Number of individuals that have specific antibodies and may still shed pathogen
			Recovered	Number of individuals that exhibit antibodies and do not shed pathogen
			Outbreak start date	Date when infections began in the site
			Birth pulse date	Date when the birth season begins
			Proportion of new susceptibles	Rate at which new births enter the host population
			Sensitivity (pathogen, antibody)	True positive rate for pathogen or antibody detection
			Specificity (pathogen, antibody)	True negative rate for pathogen or antibody detection
		Output	Percent of population (SIBR compartments)	SIBR curve over time from start of outbreak showing proportion of population in each compartment
			Proportion positive pathogen tests	Proportion of pathogen tests expected to be positive over time
			Proportion positive antibody tests	Proportion of antibody tests expected to be positive over time

*Note:* Definitions for SASSE app metrics and designation of each.

#### 2.2.1 Data Analysis Model Information

The detection and prevalence modules use the same underlying statistical model, as do their respective power analysis modules. Similar to models described in Booth et al. (2023, 2025), we leverage a Beta‐Binomial framework to account for the process that generates a “success” (i.e., presence or absence of the pathogen or antibodies); however, we add an observation process that allows for sensitivity and specificity to vary across diagnostic assays and ecological subgroups. This varies our assumptions from Booth et al. (2023, 2025) where they assume that hosts naturally cluster into social groups that share disease more readily among group members, while we assume that sampling strata represent a set of hosts that share a common likelihood of being sampled. We implement the model in a Bayesian framework, which allows users to incorporate existing knowledge on disease dynamics and diagnostic fidelity as prior distributions on parameters such as disease prevalence and sensitivity and specificity. In this model, a sampling population is a group of individuals that are equally representative of one another and can be sampled randomly. Each sample is a test result that is either positive or negative based on the underlying model. Broadly, all samples are assumed to come from a binomial distribution with a probability of disease detection shaped by two separate processes: the probability of disease presence in the population and a prior distribution on disease prevalence (i.e., what proportion of individuals are currently infected). Thus, users can account for uncertainty at two levels: whether a population is infected and what proportion of individuals are likely to be infected after an outbreak. Each sample is modeled as the result of a diagnostic test with a user‐specified prior on sensitivity and specificity, such that the possibility of inaccurate test results is explicitly included in the model. A more detailed description of the model can be found on our GitHub page (archived: https://doi.org/10.5281/zenodo.16647143 public link: https://github.com/deerdisease/sasse). Activities for each module are intended to help users build intuitions about the quantities that the model estimates (e.g., probability of disease freedom, estimated prevalence), while also developing intuitions about how the strength of prior assumptions can affect inference and uncertainty of these quantities.

#### 2.2.2 Power Study Model Information

Statistical power is the probability that an experimental design and hypothesis testing framework will correctly identify an effect at a given size and a specified false positive rate. Power studies are then formalized power analyses intended to inform experimental design. They typically answer the question: given a priori ideas about an effect size, differences among subgroups within a population, and some fixed false positive rate, what sample sizes are needed to consistently detect an effect? In a Bayesian framework, this corresponds to how credible intervals change as sample size increases. When working in wildlife management, power studies can help guide decision‐making about sampling design to ensure adequate statistical power without wasting resources on unnecessarily intensive sampling.

In the context of our disease freedom and prevalence modules, we are interested in either detecting disease at some true prevalence in the population (defined by the user within the module) or accurately estimating a true prevalence within some margin of error. In detection, this fixed prevalence value (often called a “design prevalence” in disease freedom models, Martin et al. 2007; Anderson et al. 2013) can be thought of as a hypothesis about disease prevalence. An experiment with a high probability of disease freedom would be akin to “rejecting” the hypothesis that a given disease is present at a given prevalence—in that the data collected are highly unlikely to have been generated by a system with the specified true prevalence. A power study then assesses how reliable these tests are—given a sample size and a true prevalence, how often will a disease freedom study *incorrectly claim* disease freedom (or correctly claim it if we set the true prevalence to 0)? Given our a priori knowledge of the epidemiology of a given disease, researchers can use power studies to decide what sample size is needed to consistently detect a disease with the fewest resources needed.

## 3 Module Workflow

The tools were designed for the following audiences: (1) wildlife professionals who are interested in developing skills to design, collect, or interpret disease surveillance data; (2) wildlife students interested in learning about how different ecological processes and diagnostic assays may affect study design or inference; and (3) educators interested in teaching wildlife disease surveillance principles with interactive tools that do not require advanced training in statistics.

We deployed the SASSE application and corresponding teaching materials as a pilot effort at the annual, national meeting of The Wildlife Society (TWS 2024) in a workshop titled “Wildlife disease surveillance design tools: trade‐offs between design elements.” The workshop had roughly 40 participants from diverse backgrounds, both in wildlife professions (operations, modeling, leadership, etc.) and in types of organization (academic, government, private, etc.). Skills in statistics and programming varied among participants. Workshop materials were built to deliver lessons and surveillance design insights to any wildlife audience without requiring technical experience.

The workshop agenda followed a module workflow, where each module included ecological and statistical background, an application tutorial, and a hands‐on activity; the modules were presented in progression, so that key concepts were repeated and modules were structured to build off each other. We focused on SASSE's three core modules of detection, prevalence, and epidemiological dynamics. Here, we outline an example of how we deliver each concept.

### 3.1 Module 1: Detection

The objective of this module is to help users build intuition about choosing sample sizes that lead to their desired level of confidence for disease detection in a sampling site.

#### 3.1.1 Learning Materials for Detection

Our detection module evaluates the likelihood that a pathogen is present in the sampling site, given uncertainties about how the system is sampled and the diagnostic assay performance. Specifically, this module is designed to answer: “what is the minimum number of samples required to confirm disease freedom in a population?” or “what is the expected number of samples required to detect disease?”

A unique characteristic of wildlife populations is that sampling strata are not distributed homogeneously on the landscape and vary in behavior. This means that the sources of opportunistic samples (e.g., hunter harvest, roadkill, trapping) used for surveilling the population may be biased toward observation of particular groups (nonrandom sampling), which can translate to a biased estimate of disease presence. Similarly, diagnostic assays used to detect disease in wildlife are often not optimized for use on the particular wildlife species being studied (Helman et al. 2020; Hewitt et al. 2025), such that the diagnostic results are not 100% accurate. There are two important types of error that can be introduced through diagnostic assays: sensitivity and specificity. In addition to accounting for assay sensitivity and specificity, the models used in our detection tools account for differences in probabilities of detection among sampling strata that might also be subject to differential sampling. We do this by allowing multiple “sampling stratum” entries for any process that results in nonrandom distribution of disease on the landscape and multiple “observation data” entries for any sample collection method or assay type that has differing sensitivity and specificity, all to inform the target population. Due to this stratification concept, it is important to note that when using sample size applications from Booth et al. (2023, 2025), sample sizes for declaring freedom from disease may not agree (Booth et al. 2023, 2025). Thus, users will need to choose the application that best suits the assumptions of their system (Hanley et al. 2024, 2025). For example, when dealing with grouping that comes from social or kin structure, where high contact subsets of a population may have similar probabilities of infection, Booth et al.'s (2023, 2025) formulation would capture this nonindependence well as a correlation among samples within a cluster. In situations where assays perform differently on specific sample types (e.g., live samples vs. roadkill) or where prevalence changes due to biological distinctions like developmental stage or sex, our model would allow users to define these distinct groups with independent priors on assay performance and group‐level prevalence. Different disease and surveillance systems will have different properties, and thus careful model consideration will help clarify where and why each modeling application yields different results.

Sensitivity and specificity are necessary to understand in that they can influence detection outcomes in a given sample design. We start with an introduction to the two processes in diagnostics: an individual's true disease status and the status resulting from the diagnostic assay. Sensitivity is a measure of how often a test will return a positive result when an animal is truly positive (the true positive rate), whereas 1—Sensitivity defines how often a truly positive individual tests negative (the false negative rate). Specificity measures how often a test will return a negative result when an animal is truly negative (the true negative rate), while 1—Specificity defines the false positive rate (how often a truly negative individual tests positive). These rates define how much managers can “trust” any given test result, and thus are integral to accurately estimating pathogen detection probabilities and their associated precision. We allow sensitivity and specificity to vary across both diagnostic tests and sampling strata to allow for physiological differences and differences in the method of sample procurement (e.g., diagnostic tests may have different sensitivity and specificity rates for tested samples derived from road‐killed animals as compared to animals killed via depredation permits).

In the detection module, participants use SASSE to (a) analyze data to predict disease detection and (b) decide when, where, and what quantity of samples to collect. Models to analyze detection data were implemented in a “Data Analysis” tool to provide sample interpretation outputs of the probability for disease presence/absence and upper bound for prevalence (Table 2). Models to determine sampling quotas were implemented in a “Power Study” tool that quantifies the statistical power required to detect pathogen presence under a given combination of ecological scenario and sampling design. A key input of the data analysis tool is the user's “best guess” about the probability of disease presence in the population being sampled. Statistical model assumptions for detection include (1) constant disease prevalence during the sampling event over which inference is made, (2) small sample size relative to population, and (3) all individuals in each sampling group have an equally likely chance of being sampled. Because assumption 3 will be violated in most wildlife systems when the sampling group extends across the entire site, we allow the user to introduce structure into the sampling design through “sampling strata” (defined, by sex, age, reproductive status, etc.); each group can have its own prevalence prior assigned. Additionally, sensitivity and specificity inputs can vary for each group if assays are known to perform differently. This capability enables the user to account for known sources of sampling bias that lead the surveillance design to have a higher probability of detecting the pathogen in some groups than in others.

#### 3.1.2 Application Interface for Detection

##### 3.1.2.1 Data Analysis Tool—Detection

**FIGURE 2:**
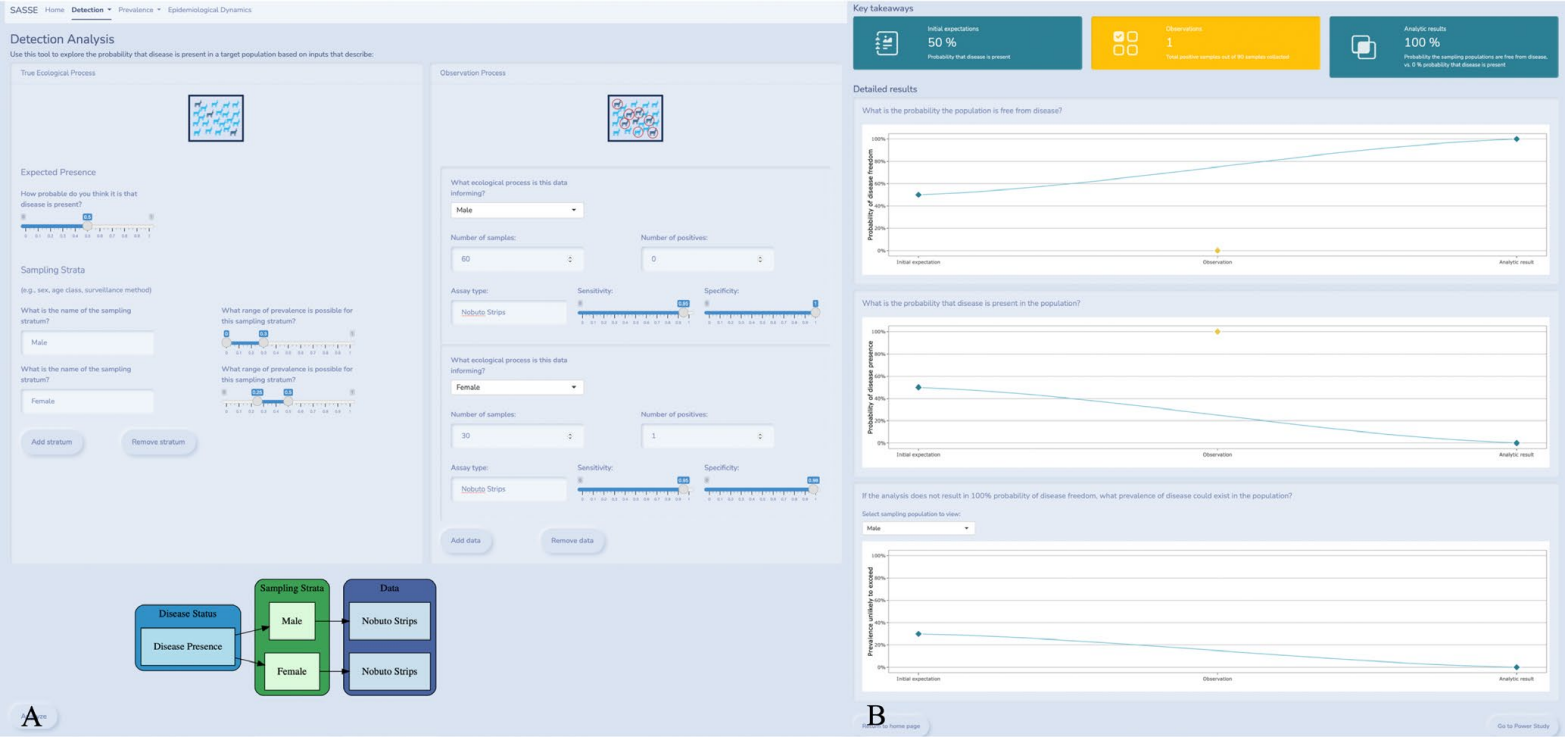
Data analysis tool. An example of the detection data analysis tool with inputs configured for two sampling strata, Male and Female, each set with a different prior prevalence range that reflects that stratum. The left panel (A) shows the inputs for the detection data analysis tool. There are inputs for the ecological process on the left and for the observation process on the right. Below the user inputs is a dynamic diagram to visualize the input configuration. Outputs are displayed in the right panel (B), which includes highlighted values in cards and the three output graphs.

Our data analysis tool allows users to iteratively construct models, input data, and view expected results based on an assumed disease process and a specified set of parameters. Ecological Process inputs include a prior probability of disease presence in the target population and an expected prevalence range for each sampling stratum within which prevalence is homogeneous. To add groups in the app, users can click the “Add Group” button and set an expected prior prevalence range (Figure 2). Under Observation Process inputs, the user can click the “Add Data” button to add rows for observed data corresponding to each sampling stratum, including number of samples, number of positives, sensitivity, and specificity. A reactive diagram beneath the user inputs then provides an image of the modified model structure (Figure 2). When the user is satisfied with the inputs, the button “Analyze” will run the model. When the model is run for one sampling stratum and one data entry, the outputs are instantaneous, whereas a more complex model with multiple sampling strata or multiple data entries will take longer to complete.

The detection analysis produces numerical and graphical outputs describing the probability of disease freedom, the probability of disease presence, and the upper prevalence bound if the analysis does not result in 100% disease freedom. Key takeaways are highlighted in cards at the top. All follow the order from left to right: Initial expectations, Observations, Analytic results. By showing all three values, we allow users to visualize how observed data and prior beliefs combine to inform the result under particular sample sizes, sensitivities, specificities, etc.

##### 3.1.2.2 Power Study—Detection

This submodule takes concepts from the data analysis tool and allows users to determine an ideal sample size for their surveillance objective through exploring the power of the surveillance design. Power study inputs include the range of possible prevalences, the probability that disease is present, and the assay sensitivity and specificity, as well as a slider for the true prevalence value. The “true prevalence” is a (hypothetical) parametric prevalence that actually governs disease dynamics; it is displayed as a slider so that it interacts with the output, allowing users to explore multiple options when assessing their study assumptions. Estimated prevalence range and probability of disease presence can be compared to that “true” value to determine the surveillance design's performance; for example, if the true prevalence is 5% in the study population, the power study module estimates how often an experimental design (i.e., sample size, assay sensitivity and specificity, and prior probability for disease presence) incorrectly declares disease freedom, or correctly declares disease presence. The power study analysis assumes experiments declare disease freedom (or presence) when experiments find at least 95% posterior probability for disease freedom (or presence). The power study analysis assumes experiments will not declare disease presence *or* freedom if both posterior probabilities are less than 95%. The power study assumptions ensure disease freedom or presence declarations are only made in the presence of statistically significant evidence. Unlike the detection analysis tool, the power study model can require several days to run all the parameter values included in the output. Therefore, a preconfigured set of parameter combinations and resulting outputs for sampling sizes from 0 to 500 is cached within the tool and can be queried for rapid results.

**FIGURE 3:**
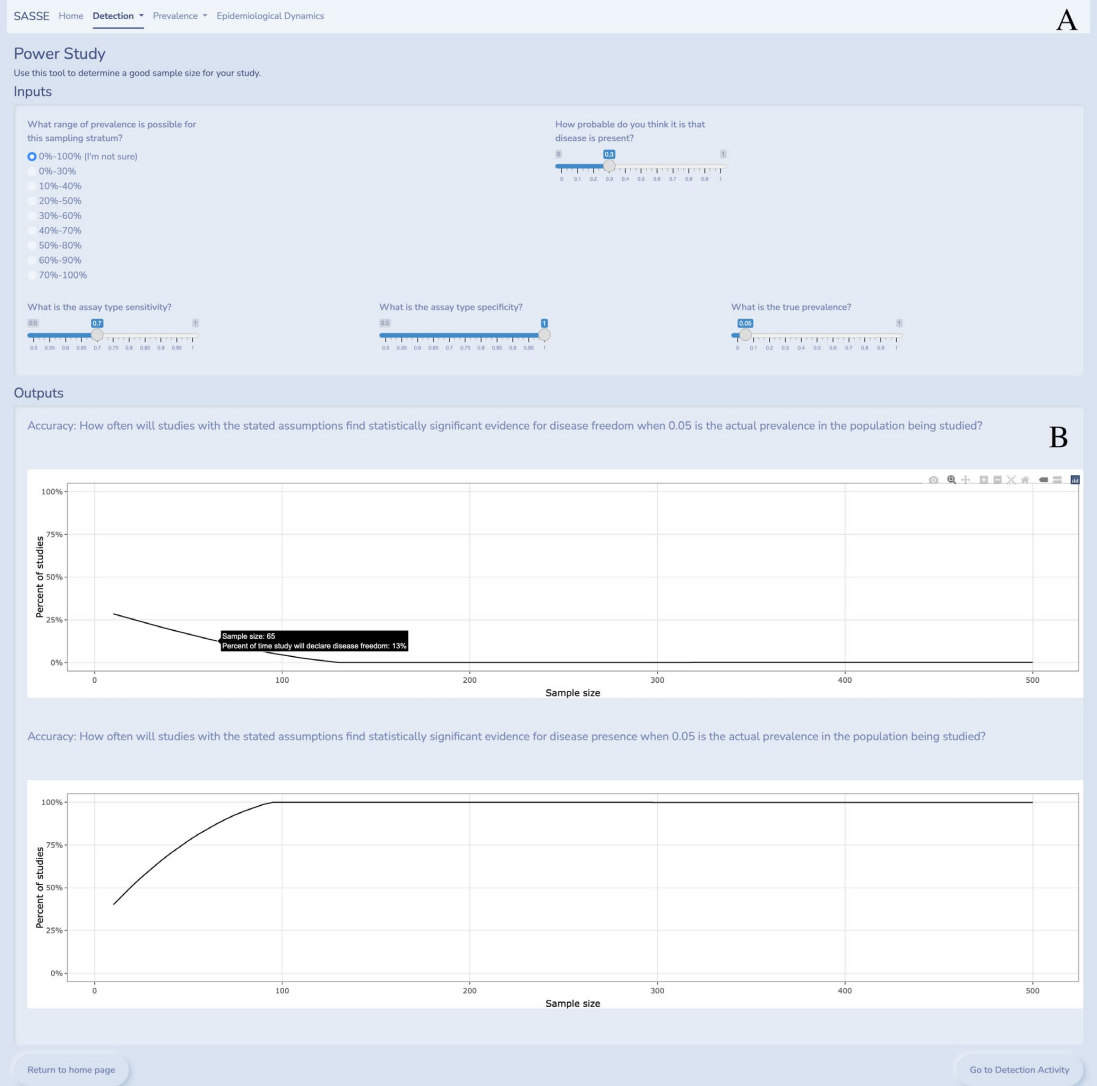
Power study module. An example view of the detection Power Study tool interface. The top panel (A) includes the user inputs, and the bottom panel (B) displays two graphical outputs.

The power study produces two interactive graphs showing sample size on the x‐axis and percent of studies (or probability) on the y‐axis. Users can place the cursor over the line on the graph to produce a tooltip showing the exact sample size and the percent of studies that will declare disease freedom (Figure 3, top panel) or the percent of studies that will declare disease presence (Figure 3, bottom panel), using the definitions above. Recall that these declarations are based on stated assumptions specified in the input section and should be compared against the “true prevalence.” This means that if the true prevalence is 0, a disease‐free declaration would be the “truth.” Users can identify the optimal sample size by hovering the mouse over the line until reaching the smallest sample size that correctly declares disease freedom is 100% (or the highest percent) of studies; if the true prevalence is greater than 0, disease presence would be “truth,” and the converse is optimal (i.e., smallest sample size that correctly declares disease presence in the highest percent of studies). If surveillance objectives are focused on disease presence, users can use the bottom graph to find the smallest sample size for the largest percent of studies with a disease presence declaration if the true prevalence is above 0 or vice versa for true prevalence of 0. Both graphs need to be considered based on surveillance objectives since studies with small sample sizes may not provide strong enough evidence to make any declaration about disease freedom or presence.

#### 3.1.3 Learning Activities for the Detection Module

Activities for each submodule of detection follow a “click, change, reflect, apply” methodology. In the first part of the activity, “click” prompts a basic question with a set of given inputs that allows the user to practice navigating the Shiny App and interpreting model outputs. “Change” sections ask users to adjust inputs to those original questions, triggering noticeable differences in the output. This allows users to connect changes in surveillance design to changes in results and interpretation. The “reflect” section builds on examples from the “click” and “change” sections to elicit a longer conceptual response. In “apply,” we ask users to consider how these ideas apply to their own surveillance system or other real‐life instances. In Text S1, section 1, we walk through an example activity for detection. Additional activities can be found on our GitHub page (archived: https://doi.org/10.5281/zenodo.16647143 public link: https://github.com/deerdisease/sasse).

### 3.2 Module 2: Prevalence

The objective of the prevalence module is to estimate true prevalence given the sampling design—including surveillance method, sample size, and test detection errors—and to understand two relevant sources of bias, confirmation bias and sampling bias. This module builds off the detection module to answer questions on prevalence and the accuracy and precision of our prevalence estimates. Accuracy refers to how close an estimated prevalence value is to the actual true prevalence in the population. Precision measures the consistency or repeatability of the prevalence estimates across repeated samples or studies, represented by the narrowness of the credible interval. A credible interval itself is a statistical range indicating where the true prevalence likely falls, given observed data and prior assumptions. It is a common measure of uncertainty in Bayesian statistics. When little is known about the true prevalence (i.e., prior is uninformative), the model reflects apparent prevalence.

#### 3.2.1 Learning Materials for Prevalence

True prevalence is the percent of individuals in the population that are infected with a pathogen at the time of sampling. True prevalence can be challenging to estimate because it requires collecting a representative sample of the population, which is often unavailable. Thus, surveillance programs often estimate sample prevalence (apparent prevalence: the percent of individuals in the sample infected with the pathogen) as a proxy for true prevalence. Apparent prevalence reflects the raw counts of what was observed in the sample and can lead to biased estimates of prevalence when sample design (how individuals are chosen for sampling) is not considered. An example of this is seen in opportunistic sampling, a common method used in wildlife surveillance frameworks, to capture baseline information about population prevalence. In opportunistic designs, sampling occurs alongside other management objectives. These objectives, for example, wildlife damage management, may target particular individuals or groups that are not representative of the entire population. If infection rates are higher in animals targeted by the focal activity (e.g., wildlife damage management) than in animals in the overall population, then apparent prevalence will be an overestimate of true prevalence. Some of this bias can be removed through statistical adjustments (e.g., Wilber et al. 2020), but ideally, the surveillance design should be built around prevalence objectives per se; once we know a disease is present, we need to understand the proportion of individuals affected in the entire population of interest. Designing surveillance around estimating true prevalence can also inform resource optimization for accuracy (credible interval includes true prevalence) and precision (credible interval width) goals, progression of an outbreak, and confidence of specific thresholds for disease monitoring and intervention strategies. When sampling is costly or labor‐intensive, surveillance programs may need to evaluate how the precision of an estimate changes as sample size increases to optimize costs of sampling alongside the benefits of increased information.

The models in the prevalence module follow the same assumptions and inputs as those in the detection module, with slight differences in how they are treated. This module assumes that pathogen presence has already been established, so the prior on disease presence should now account for that. Priors on expected prevalence can have a large impact on prevalence model outcomes, potentially leading to confirmation bias, where prevalence estimates favor prior beliefs. To set up the prevalence range for an “uninformative” model structure, users can leave the range from 0% to 100% to allow all possibilities to be equally likely. Prevalence can also be subject to sampling bias if different sampling strata have different prevalences and groups are not sampled in proportion to their occurrence within the population. Activities in this section explore how failing to account for these groups can provide false interpretation of surveillance results relative to prevalence.

#### 3.2.2 Application Interface for Prevalence

##### 3.2.2.1 Data Analysis Tool—Prevalence

Our data analysis tool for prevalence expands on the data analysis tool for detection. Inputs include priors on disease presence and prevalence range (ecological process), data entry for number of samples and number of positives (observation process), and data entry for diagnostic test sensitivity and specificity (observation process). Consequently, the user interface resembles the interface described for the Data Analysis Tool for detection. Inputs are structured with the same format, allowing the user to enter priors for the true ecological process on the left and the observation process on the right. Where the prevalence module differs from the detection module is in how the prior on disease presence is addressed: the user input for prior probability of disease presence should be set to 100% in the prevalence module because we now assume that disease is present in the population. Prevalence analysis can be run for different priors on disease presence, but this is the most common scenario. Once the model inputs are configured with appropriate priors, data, and sampling strata, the user can hit “Analyze” to run the model.

Model outputs for the prevalence module are the prevalence estimate and a corresponding 95% posterior credible interval. The presentation of the prevalence results is similar to detection results, with both graphical output and value cards highlighting key takeaways for the activities. Cards above the graph match the three points on the graph: Initial expectations, Observations, and Analytic results. A separate estimate is produced for each sampling population described in the inputs, that is, the module generates a separate prevalence estimate for each sampling stratum. Users can view results for each of their specified sampling strata through the “which sampling population would you like to view results for?” drop‐down menu. For the selected sampling population, the “Initial expectation” shows the prior expected prevalence range (bounds) and mean (point) that is possible, the “Observation” shows the apparent prevalence (point), or the raw percentage of tests that were positive, and the “Analytic result” shows the model estimated true prevalence (point) with the 95% credible interval (bounds) for that estimate. These outputs show users how the prevalence estimates reflect both the prior expectations and the observed data, and how the width of the resulting credible intervals changes based on how much data were collected, what priors were used, and values of test sensitivity and specificity.

##### 3.2.2.2 Power Study—Prevalence

The Power Study tool for prevalence builds on the prevalence concepts taught in the data analysis tool to provide users with intuition on designing surveillance that meets prevalence objectives. The prevalence power study inputs are the same as the detection power study: the range of possible prevalences, how probable it is that the disease is present (defaulted to 1), and the assay sensitivity and specificity. The slider for “true prevalence” is reflected in the model outcome, in which the outputs shown are for values centered around the “true prevalence” value. For example, the graph of “average credible interval width” shows the value for the average credible interval width for that true prevalence value. Once inputs are selected, this power study tool will repeat the same methods for under‐the‐hood computation as outlined in the power study submodule of detection Section 3.1.2 to analyze sample sizes ranging from 0 to 500.

Prevalence power study outputs include the probability that the credible interval covers the true prevalence (accuracy, top graph) and the average credible interval width (precision, bottom graph). Both outputs are displayed in interactive graphs showing the sample size on the x‐axis and the result value on the y‐axis with a tooltip option to hover over exact values. The output labeled “probability the credible interval covers the true prevalence” tells users how probable it is that a study with the proposed study design will produce credible intervals that actually contain the population's true prevalence. The question “how often will studies with the stated assumptions have a 95% credible interval that is correct when 5% is the actual prevalence in the population being studied?” is printed above the graphical output to remind users about interpretation. For this output, a greater value for “coverage probability” indicates accuracy is better for that corresponding sample size. The second output of the average credible interval width quantifies the amount of uncertainty in a prevalence estimate using a 95% credible interval. For example, if prevalence is estimated at 35% with a credible interval of 15%–45%, then the credible interval width is 45% − 15% = 30%. A wider credible interval indicates a larger amount of uncertainty, so the user should determine sample size based on an a priori target average credible interval width.

#### 3.2.3 Learning Activities for the Prevalence Module

Activities for prevalence follow the same “click, change, reflect, apply” methodology as the activities for detection. The application interface for the two submodules of prevalence aligns with the interface for the detection submodules, and therefore, the audience will typically move through the prevalence activities at a quicker pace with fewer questions on how to use the tool. We outline an example of a prevalence activity in Text S1, section 2.

### 3.3 Module 3: Epidemiological Dynamics

The objective of this module is to run a basic epidemiological model to learn how disease spreads in a population and build intuition about when and how much to sample at a site if the objective is to understand epidemiological dynamics such as seasonal changes in prevalence, length of infectious or immunity periods, or R_0_.

#### 3.3.1 Learning Materials for Epidemiological Dynamics

In the first two modules, we evaluate detection and prevalence as point estimates, but we do not discuss spatiotemporal change. Epidemiological dynamics acknowledge that pathogen prevalence in a population will vary depending on the transmission patterns and time since pathogen introduction. Compartmental models are common mathematical models to describe how individuals move from one disease state, or “compartment,” to another over time. Compartmental models classically consist of susceptible (“S”), infected (“I”), and recovered (“R”) states; however, the specific compartments used vary across wildlife disease systems, and models should be tailored to the focal system. For example, diseases with pathogen clearance but no protective immunity could be modeled with an SIS, or Susceptible‐Infectious‐Susceptible, model; diseases with a latent, noninfectious incubation period could be modeled using SEIR, or Susceptible‐Exposed‐Infectious‐Recovered; and systems where the pathogen has already established might be at an endemic equilibrium where the proportion of the population in each state does not change substantially through time. Here, we focus on teaching an example of how to use and interpret one type of epidemiological model and provide a workflow for how to use that information in the prevalence module to determine an optimal sampling approach. We expect that users will eventually apply this information to a compartmental model that is appropriate for their focal system.

Our epidemiological model used in the tutorial is an SIBR model, or Susceptible‐Infectious‐Broadly Recovered‐Recovered (Hewitt et al. 2024). This corresponds to systems such as SARS‐CoV‐2, where individuals recovering from the infection can have detectable antibodies to the pathogen while still maintaining a detectable level of virus when they are no longer infectious (Chandler et al. 2021). Disease statuses then stem from paired diagnostic assays that look at both virus and antibody presence. Other systems may lend themselves to other class definitions depending on both their underlying biology and the available diagnostic assays.

In the SIBR model, individuals move from susceptible to infectious based on the force of infection (the product of transmission, the number of susceptible individuals, and the number of infective individuals). The average infectious Days parameter defines the rate for movement from the infectious to the recovered class. Individuals then move into the “broadly recovered” class, where they remain based on a rate derived from the average recovery days (also user‐specified). Next, they move to the recovered class, where they remain for the average waning days (also user‐specified), at which point they move back to the susceptible class. The model thus assumes temporary immunity in the recovered class. The model also accommodates a user‐defined start date for the outbreak and an annual birth pulse date (Table 2).

Similar to other compartment models, the SIBR model assumes that (a) all animals are in exactly one of the disease states at every point in time, (b) the population is well‐mixed in the sampling site, and (c) changes across time are adequately described using differential equations. This model assumes that recruitment of new susceptible animals occurs as an annual birth pulse, similar to many wildlife species, with the timing of that pulse defined by the user. The user must input transition rates between disease compartments, birth pulse sizes and timings, initial compartment sizes, and sensitivity and specificity for pathogen and antibody tests. The model then returns the proportion of the population in compartments over time and the expected positivity rates for pathogen and antibody tests. This module helps to develop intuition for how the numbers of individuals in each disease state may change over time, with interpretation for changes in sample sizes.

#### 3.3.2 Application Interface for Epidemiological Dynamics

**FIGURE 4:**
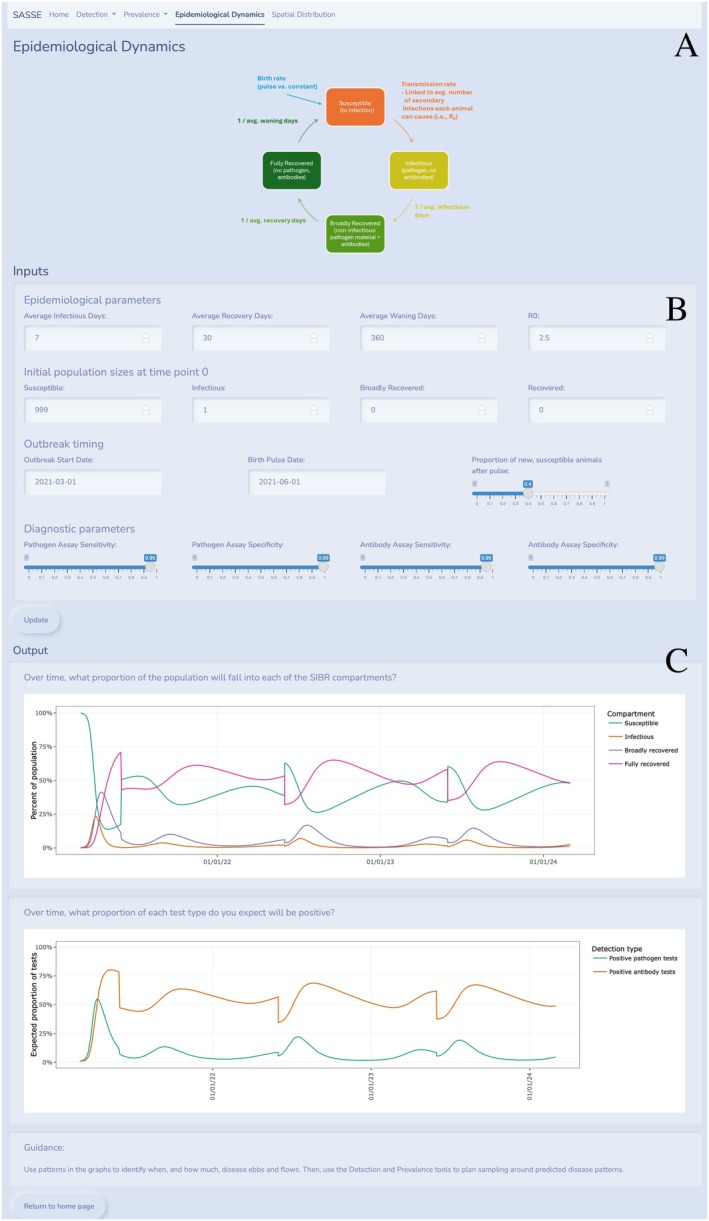
Epidemiological dynamics module page. An example of the interface for the epidemiological dynamics module. In the top panel (A), a diagram of the SIBR model is displayed as a visual aid to the users. The middle panel (B) contains all of the user inputs described in Section 3.3.2: Application interface for epidemiological dynamics. The bottom panel (C) contains two output graphs: one showing the percent of the population in each SIBR component, and one showing the proportion of positive tests for pathogen tests and antibody tests.

Our epidemiological dynamics tool lets the user run an epidemiological model and identify patterns to inform their surveillance design. Compartmental models can be used in several ways, depending on the focal disease system, but here we demonstrate epidemiological dynamics using a SIBR model as our default. A diagram of the SIBR components is displayed at the top of this module as a visual aid (Figure 4). Inputs for this module are broken into four categories: (1) epidemiological parameters, (2) initial population sizes at time point 0, (3) outbreak timing, and (4) diagnostic parameters. Epidemiological parameter inputs are all numerical entry boxes and include average infectious days, average number of days that individuals are immune but not infectious while still shedding detectable levels of virus, average number of days until immunity wanes from detectable levels of antibodies and immunity to infection, and R_0_ (defined in Table 2). The user provides the number of individuals in each disease category at time 0 as numerical inputs with the unit being an individual, and default disease states include susceptible, infectious, broadly recovered, and recovered. Outbreak timing inputs include the outbreak start date, the birth pulse date, and the proportion of new, susceptible animals after the birth pulse. Currently, there is only an entry for one birth pulse, but a feature to add multiple birth pulses is under development. Diagnostic parameter inputs include slider bars for pathogen and antibody assays. Once the user has filled in the epidemiological model inputs, the “update” button renders output graphs.

Outputs include two graphs and a statement to help users apply information from the curves to the detection and prevalence modules to inform sample planning. The first graph (Figure 4, top panel) is the SIBR curve plotted over 3 years from the user‐specified outbreak start date. This graph can help users determine, “over time, what proportion of the population will fall into each of the SIBR compartments?” Each disease compartment is represented by a line colored in the legend, with an interactive mouse‐over tool that allows users to view the compartment, date, and percent of the population in that compartment along each line. The second graph (Figure 4, bottom panel) helps the user determine, “over time, what proportion of each test type do you expect will be positive?” This graph is plotted over the same time range as the first and displays two lines representing detection type—positive antibody tests and pathogen tests. Tooltip pop‐ups show the date, expected proportion of positive tests, and detection type for points along the two lines. Interpretation of the two graphs should prompt users to consider patterns in the graphs to identify when, and how much, disease ebbs and flows to then use the detection and prevalence tools to plan sampling around predicted disease patterns. To do this, users can look at the compartmental model in terms of surveillance time points and then use the associated proportion of tests graph to create priors for prevalence range in the detection and prevalence module. This can be used to determine how much data would be needed for a desired level of accuracy.

#### 3.3.3 Activities for Epidemiological Dynamics

Activities for epidemiological dynamics follow the same “click, change, reflect, apply” methodology as the detection and prevalence. This module differs in that the interpretation process involves taking outputs from this module to use as priors in the detection and prevalence modules for specific sampling guidelines. We provide an example of how to walk through an activity in this module in Text S1, section 3.

## 4 Discussion

We provide a broadly accessible tool for planning statistically grounded disease surveillance in wildlife systems (Drewe et al. 2012), in a format that promotes education and development of intuition for designing statistically sound surveillance designs (Nusser et al. 2008). Our tool adapts existing livestock methods (Artois et al. 2001; Cardoso et al. 2022) for application in wildlife disease surveillance. SASSE considers: (1) program objectives, that is, determination of disease presence, prevalence, or growth (Cheng et al. 2020; Nichols et al. 2021), (2) stage in disease emergence, for example, endemic, emerging, or re‐emergent (Thulke et al. 2009; Clow et al. 2019; Wilber et al. 2020; Walton et al. 2016), and (3) availability of financial, human, and information resources (Clement et al. 2023; Stärk et al. 2006; Guberti and Newman 2007; Shwiff et al. 2016) in wildlife systems. Thus, SASSE delivers insight on optimization of wildlife disease surveillance design through interactive exploration of resource trade‐offs in different surveillance system contexts (stages and objectives).

### 4.1 Application of SASSE

This manuscript documents the teaching materials we developed for the pilot deployment of the SASSE application, where we evaluated our tool and its delivery in a teaching environment. Our pilot workshop and practice sessions with other staff highlighted considerations for technology support when a large number of users open the same online application simultaneously in the classroom. Following these experiences, we optimized the code for use with multiple concurrent users and distributed the application through multiple hosting methods (downloadable from GitHub and Shiny.io) to accommodate the use of SASSE for a larger volume of users.

In addition to the wildlife education/academia audience demonstrated, we propose that SASSE is beneficial for wildlife professionals and operations personnel in surveillance programs. SASSE can be used for rapid sample size planning or inference about disease presence from field data. For example, in situations where data collection is ongoing, SASSE can provide an accessible, interactive framework for incorporating novel data into surveillance design for general guidance on sample size and power to detect disease or infer a given prevalence. In other scenarios, where data may be available but sparse, SASSE's Bayesian approach could allow managers to explore how uncertainty in critical parameters changes surveillance needs. These data situations are common in wildlife disease surveillance programs that are (1) in the start of an outbreak where data has not yet been collected and the program needs some insight on design given priors, (2) during an outbreak where data are coming in and the program would like quick guidance on progress or refined design recommendations based on new data, or (3) monitoring endemic diseases where the host density is unknown and the program needs estimates given a lack of data.

### 4.2 Extended Applications

The current version of SASSE focuses on surveillance objectives of detection, prevalence, and epidemiological dynamics, but we expand our education material to answer questions on seasonality. To use this tool for seasonality interpretation, we introduce the concepts of sampling strata changing over time and how to approximate the changes with sampling design. We discuss decision‐making around selecting timing for and frequency of sampling periods. Using the existing tools, users are able to take the detection and prevalence tools and use the “sampling strata” functionality to represent different time periods—for example, if looking at two seasons, inputs would be configured to have a group for the “peak season” and a group for the “low season” each with different input parameters describing the population at those time points. Thus, the tool output can be used to plan how to optimize sample sizes across seasons within budget constraints or annual sampling quotas. Currently, this is done separately for each season (sampling occasion) by identifying effective sample sizes in peak and nonpeak seasons and manually deciding how many different seasons a theoretical total sample quota will be spread across. More complex analyses could be developed into the model and application interface to display effective sample sizes over time for a user‐specified number of sampling seasons.

### 4.3 Future Development

A common surveillance objective that our tool does not address explicitly is to determine the spatial distribution of a wildlife disease. Spatial distribution can be addressed through leveraging our detection module for application across some predefined spatial extent—for example, understanding tradeoffs between the number of sites and sample size at each site while maintaining confident detection ability.

Our tool could also be used for integration in a larger adaptive surveillance framework. The concept of adaptive management has been proposed to address common challenges in wildlife surveillance through objective‐driven optimization of spatial and temporal resource allocation with iterative data integration to improve learning about the disease system (Williams and Brown 2016; Miller and Pepin 2019; Pepin et al. 2021; Miller et al. 2022). There is a wealth of literature suggesting and describing the need for context‐specific surveillance plans, designs based on program objectives, or adaptive risk‐based surveillance approaches (Peyre et al. 2019; Cheng et al. 2020; Cardoso et al. 2022; Clement et al. 2023; Cameron 2012; Cameron et al. 2014; Clow et al. 2019; Miller et al. 2022; Thulke et al. 2009). SASSE, in its current version, is built around the objective‐based decision‐making concept with outputs focused on changing uncertainty for different surveillance designs, providing insight into the answers to the fundamental questions asked in adaptive surveillance. To meet the second piece of an adaptive framework, the ability to “adapt” design recommendations to ongoing data collection and changes in the system, SASSE can be used iteratively to update and refine inputs from new information—for example, a starting point could have a wide range for the expected prevalence slider and, as the user learns more from the first round of surveillance data, the slider may decrease in width and have a more specific idea of what the prevalence prior should be for the population, which would then provide updated sample size and power outputs. Although SASSE, as a stand‐alone tool, is able to provide some rapid insight into key pieces of adaptive surveillance, it could still be expanded upon for a more robust plan. For example, automating adaptive surveillance across surveillance seasons through integrating a spatial risk assessment module that uses historical surveillance data linked to user‐selected covariates. This module could provide visualization for identifying high‐risk locations and sets of risk factors for targeting future surveillance, whereas the sample size modules could be used to plan sample sizes in the target areas. Automating visualizations of risk assessment would deliver rapid epidemiological intelligence to stakeholders for planning biosecurity while improving accessibility of rigorous surveillance design tools for increasing the efficiency of surveillance programs.

SASSE tool materials were designed openly to allow other researchers to collaborate on further development for addressing the needs of wildlife disease students, educators, and professionals. All learning materials, model guidelines, and code are publicly available on our GitHub (archived: https://doi.org/10.5281/zenodo.16647143 public link: https://github.com/deerdisease/sasse).

## Author Contributions

**Lauren Smith:** conceptualization (equal), methodology (equal), software (lead), visualization (lead), writing – original draft (lead), writing – review and editing (equal). **Joshua Hewitt:** conceptualization (equal), methodology (lead), software (supporting), visualization (equal), writing – review and editing (equal). **Aaron Westmoreland:** methodology (supporting), software (supporting), writing – original draft (supporting), writing – review and editing (equal). **Grete Wilson‐Henjum:** conceptualization (supporting), methodology (supporting), validation (supporting), writing – review and editing (equal). **Kezia Manlove:** writing – original draft (supporting), writing – review and editing (equal). **Kim M. Pepin:** conceptualization (equal), funding acquisition (lead), methodology (supporting), supervision (lead), writing – review and editing (equal).

## Conflicts of Interest

The authors declare no conflicts of interest.

## Supporting information


**Data S1:** ece371991‐sup‐0001‐Supinfo1.docx.

## Data Availability

All data and code for reproducing our Shiny App described in this manuscript are made publicly available on our GitHub (archived: https://doi.org/10.5281/zenodo.16647143 public link: https://github.com/deerdisease/sasse). To run the “Power Study” module of SASSE, code to generate the simulation data is contained in the GitHub repository, or an example dataset can be downloaded from: https://doi.org/10.5281/zenodo.16646282. Link to our public‐facing tool (https://deerdisease.shinyapps.io/Wildlife‐surveillance‐design‐tools) and DOI for our GitHub (https://doi.org/10.5281/zenodo.16647143) page are embedded throughout the main text.
