# Supplementary material for: Surveillance Analysis and Sample Size Explorer (SASSE): Learning How to Plan Disease Surveillance in Wildlife
Source: Ecol Evol. 2025 Aug 15;15(8):e71991. doi: 10.1002/ece3.71991 (PMC12356644; doi:10.1002/ece3.71991)
Supplement: Supplementary file 1 — Data S1: ece371991‐sup‐0001‐Supinfo1.docx. [file ECE3-15-e71991-s001.docx]

**Supplementary Text S1: Example Activities**

**Section 1.** Example activity for detection analysis

In the ‘click’ question, we build basic understanding on the disease freedom metric and the maximum prevalence metric by asking ‘What is the probability the population is free from disease if a) 30% of populations tend to have disease, b) infected groups tend to have 1-15% prevalence, c) you have 0 positive test results across 30 samples from this population, and d) the diagnostic test has 70% sensitivity and 100% specificity?’ The user should find strong evidence that there is either no or very low disease, with a 92% probability of disease freedom and prevalence not likely to exceed 5% otherwise. Next, the ‘change’ question asks, ‘Starting with inputs from the initial prompt, a) how many samples do you need to get a 99% probability of disease freedom, and b) how does that probability change if you have 1 positive sample out of 30 total?’ Users should find that at least 79 samples are required for 99% probability of disease freedom, and that the probability changes to 0% once there is one positive sample.

This section should help users connect experimental design with conceptual interpretation and recognize 1) how to change sample size to reach desired metrics, and 2) how negative and positive test results change model outputs. The ‘reflect’ question asks users to consider how the detection metrics depend on sensitivity and specificity when there is a positive test result versus no positive test results. Users conclude from this activity that: 1) specificity (false positive rate) and sensitivity (false negative rate) provide information on confidence in our sample results, 2) specificity is very important when there is at least one positive test result, and 3) sensitivity has a larger impact than specificity when there are no positive test results. This is important in surveillance design planning because better test sensitivities and specificities will require smaller sample sizes to reach the same level of surveillance program certainty. In wildlife examples involving sampling strata, these concepts can be applied to optimize design around group-specific test values.

Finally, the ‘apply’ question asks how probabilities of disease freedom and presence and estimated prevalence depend on sample size and disease dynamics, and prompts users to consider application to their surveillance design. Users should recognize that 1) sample size has a big impact on disease freedom probability when specificity is less than 100% and 2) priors for disease (i.e., expected presence and possible prevalence range) impact disease freedom probability more when sample size is small. Then, we discuss how to translate results to wildlife management actions and draw connections to the disease priors. In designing surveillance, we consider impacts of sampling quantity and timing on the ability to detect disease when present on a landscape and how recommendations change based on if detection is the primary goal. Our recommendations often align with livestock models where we aim to demonstrate disease freedom at a given prevalence (design prevalence) to, for example, show that a farm or county is safe for trade. In our case, we might use ideas about seasonal prevalence to inform a range of ‘design prevalences’ and then establish how likely it is that the disease is present within that range.

**Section 2.** Example activity for prevalence analysis

Starting with ‘click’, we ask, ‘What will the prevalence estimate be if a) it is known with 100% certainty that disease is present in the population, b) we are unsure if prevalence is currently large or small, c) we have 25 positive test results from 50 total samples collected from this population, and d) the diagnostic test has 95% sensitivity and 95% specificity?’ The user should find that prevalence is estimated to be 50% with a 95% credible interval of 35-65%. Here the user should note that because the prevalence prior is uninformative, the model estimate reflects the apparent prevalence. Next, we have two different pairs of ‘change’ and ‘reflect’ questions that each present a significant takeaway:

Path A Change – ‘Instead, you have strong initial beliefs or expectations that prevalence is small, say between 0% and 15%.’ In this case, the prevalence estimate is 26% with a 17-35% credible interval. Users will see that the prior has a large impact and what happens when observed data is not within the prior prevalence range. ‘Reflect’ asks how these estimates depend on sample size and prior belief. Main points here should be that prevalence estimates are heavily influenced by strong prior assumptions when sample size is small, and thus larger sample sizes are needed to avoid confirmation bias.

Path B Change – ‘Actually, while you do in fact have 50 samples with 25 positives, 40 of those are males (which typically have a prevalence of 30%-70%) and 10 are females (which typically have a prevalence of 0%-15%). 24 of our positive samples are males and only 1 is a female. Recalculate prevalence for each group accounting for differences in priors (this may take a few minutes).’ Users should find that males have 57% prevalence with 43-69% credible interval and females have 8% prevalence with 3-15% credible interval. ‘Reflect’ should teach the user how accounting for differences among sampling strata can improve estimates of population prevalence as a main teaching point.

Wrapping up the activity with ‘apply’, we ask attendees to consider the two types of surveillance design biases described in this activity. We ask, ‘how can surveillance programs mitigate this bias or consider it in their surveillance design or management decisions?’ User answers will vary, but in general, should evoke discussion on ideas for structuring surveillance design to avoid confirmation bias and sampling bias as best as possible.

**Section 3.** Example activity for epidemiological dynamics

We first ask a click question, ‘What is the maximum proportion of the population that will test positive for a pathogen in the first three years, and when do the maximums occur if a) animals remain infectious for 7 days, on average [Average Infectious Days], b) immune systems remove all infectious material in 90 days, on average [Average Recovery Days], c) individual immunity lasts one year, on average [Average Waning Days], d) each infectious animal infects 2.5 additional animals, on average [R_0_], e) initially, 999 individuals in the population are susceptible and 1 is infectious [Initial population sizes at time point 0], f) the first infection occurred on 3/1/2021 [Outbreak Start Date], g) 40% of total population is new, susceptible animals after spring births (i.e., 6/1) [birth pulse inputs], and h) sensitivity for the pathogen test is 95%, and specificity is 99%?’ Users should answer with 69% on 4/16/2021, 38% on 7/25/2022, and 8% on 1/1/2023. Next, the ‘change’ question asks users to instead have individual immunity last 18 months on average and show that each infectious animal infects 1.5 additional animals on average. The updated answer should be 33% on 5/30/2021, 28% on 7/12/2021, 6% on 1/1/2022, and 1% on 2/8/2023. These first two questions focus on extracting values from the second graph (Figure 4, bottom panel) but should guide users through model input changes that can be visualized in both graphs to build intuition on how epidemiological models can change based on inputs.

‘Reflect’ asks ‘How can epidemiological dynamics potentially impact surveillance plans?’ This guides discussion on how small changes to disease parameters can have a large impact on disease dynamics, e.g., instead of regular annual peaks in prevalence, multi-year disease patterns can be more dispersed in timing and scale. We discuss this as a challenge in wildlife surveillance systems as estimating epidemiological parameters can be challenging. Wildlife surveillance systems should consider how planning may need to adapt timing and effort from year to year as we refine epidemiological parameters. Another conclusion is that maximum test positivity may not coincide with maximum infectiousness. We wrap up this activity with an ‘apply’ question which asks ‘What parameters have the greatest impact on peak disease timing and consistency? How can you design a surveillance system to accommodate the impacts?’ Exploration of the epidemiological dynamics tool should lead to users finding immunity duration, transmission rates, and birth pulse timings to have the greatest impact on peak disease timing and consistency. Major takeaways from this discussion should be that surveillance plans need to cover a range of potential minimum and maximum prevalence times to help ensure adequate monitoring at all epidemic stages.

In the absence of a seasonality module, this activity for epidemiological dynamics ends with drawing connections to seasonality interpretation. This includes evaluating a target window with peaks and valleys to get a good picture of seasonality when deciding on how to aggregate the collection time frame i.e., how wide the time period or season should be. Further applications of seasonality are described in the lecture content on GitHub (https://github.com/deerdisease/sasse).
